# Surgical Management of Iliopsoas Impingement With Combined Acetabular Revision and Partial Psoas Tenotomy

**DOI:** 10.7759/cureus.13193

**Published:** 2021-02-07

**Authors:** Andrew Yun, Marilena Qutami, Kory B Pasko

**Affiliations:** 1 Orthopaedic Surgery, Center for Hip and Knee Replacement, Providence Saint John’s Health Center, Santa Monica, USA; 2 Orthopaedic Surgery, Georgetown University School of Medicine, Georgetown Hospital, Washington, DC, USA

**Keywords:** iliopsoas impingement, revision total hip arthroplasty, acetabular retroversion, diagnostic injection, direct anterior approach, hip replacement, total hip replacement, total hip arthroplasty

## Abstract

Background

Persistent groin pain after total hip arthroplasty (THA) can result from iliopsoas impingement (IPI) on the acetabular rim. Controversy exists over the risks and benefits of tenotomy versus revision as a surgical solution. We report our limited experience with combined acetabular revision and partial iliopsoas tenotomy when other conservative treatments have failed.

Methodology

A total of eight patients revised for IPI by a single surgeon at a single institution were retrospectively reviewed after a minimum one-year follow-up. Preoperatively, all patients had prolonged groin pain for a mean of two years (range: 1-4 years) and had failed conservative treatment for at least six months. All patients underwent acetabular revision through a direct anterior approach (DAA) with partial psoas tendon release. No stems were revised. Dislocations, complications, and clinical outcomes are reported in this study.

Results

Of the eight patients, seven had a positive diagnostic challenge with an image-guided injection. All revised cups showed radiographic evidence of IPI with relative acetabular retroversion by either a cross-table lateral film or computed tomography scan. Preoperatively, the mean cup anteversion was 4 degrees (range: 0-9 degrees). Postoperatively, the mean cup anteversion was 19 degrees (range: 16-21 degrees). All cups were within the so-called safe zone. To avoid overstuffing, the mean cup size remained unchanged. There were no major postoperative complications. At a mean time to follow-up of 3.3 years, the mean Hip disability and Osteoarthritis Outcome Score for Joint Replacement was 75 points (range: 32-100 points).

Conclusion

IPI may be effectively managed with combined acetabular revision and tenotomy. The challenges of implant placement and positioning may be aided with intraoperative imaging through a DAA THA.

## Introduction

Iliopsoas impingement (IPI) after total hip arthroplasty (THA) remains a challenging complication in terms of diagnosis and treatment. Although it is underrecognized as a cause of persistent groin pain, its incidence has been reported to be as high as 4.3% in a targeted series [1]. While the clinical symptoms have been described as sometimes “subtle,” patients with refractory IPI may demonstrate overall poor outcomes with reported Harris Hip Scores as low as 50 to 60 points [2,3]. Anatomically, the mechanical overhang of the acetabular construct impinges on the iliopsoas tendon during hip flexion. Implant malposition, cup retroversion, protruding screws, and excessive lateralization have all been implicated as risk factors in cementless constructs.

Treatment may be broadly divided into conservative and surgical options. Conservative options including physical therapy, anti-inflammatories, and tendon sheath injections have met with limited success. Their utility in the management of IPI may be more helpful diagnostically than therapeutically. Alternatively, surgical treatment has centered on iliopsoas tenotomy or acetabular cup revision. The outcome of correcting IPI with acetabular revision has been reported to be successful in relieving symptoms but is also marred at times by a complication rate as high as 19.4% [2]. In contrast, open iliopsoas tenotomy without acetabular revision is associated with pain relief in 75% of the patients with a 33% complication rate [4].

An alternative technique for revision total hip arthroplasty (rTHA) was developed utilizing the direct anterior approach (DAA) and intraoperative imaging. As the primary pathology in IPI is located at the anterior hip, it seemed reasonable to approach the problem from the front. In this anatomic window, both cup position and tendon insertion are readily accessible. Moreover, fluoroscopy can serve as a useful adjunct in correcting cup malposition. In this paper, we present the technique of isolated cup revision with partial iliopsoas tenotomy for IPI. We examine the complications, perioperative course, and long-term outcomes of rTHA using the DAA.

## Materials and methods

We retrospectively reviewed a total of eight patients with IPI who underwent rTHA with partial tenotomy. A DAA with intraoperative fluoroscopy was used in all cases. Surgery was performed by a single surgeon at a single institution with a minimum of one-year follow-up.

**Figure 1:**
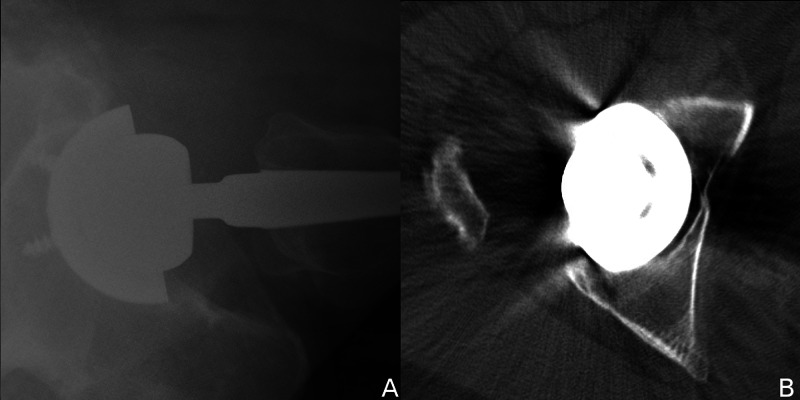
Radiographic diagnosis of IPI. (A) Cross-table lateral radiograph with anterior cup prominence from relative retroversion. (B) Axial CT scan of the right hip with anterior rim of the cup extending beyond the anterior wall of the acetabulum. IPI, iliopsoas impingement; CT, computed tomography

All patients failed an extended period of conservative treatment of at least six months. The diagnosis of IPI was confirmed by a combination of examination, diagnostic iliopsoas tendon sheath injection, and imaging. Clear evidence of anterior cup protrusion beyond the pelvic rim was noted on either a cross-table lateral radiograph or a computed tomography (CT) scan with reconstructions [Figure 1]. Other potential causes of groin pain including aseptic loosening, instability, and infection were ruled out.

Only patients with acetabular cup, liner, and femoral head exchange were included. No stems were revised. Charts were reviewed for indications, clinical history, and prior treatment failures. The primary outcome measures were acetabular size and position, intraoperative or postoperative complications, and the need for revision-type implants like cages, augments, or allografts. Acetabular bone loss after cup removal was graded with the Paprosky Classification [5]. Secondarily, records were reviewed for hospital course, reoperations, and readmissions. Clinical outcomes measured were patient-reported Hip disability and Osteoarthritis Outcome Score for Joint Replacement (HOOS, Jr), revision, and death [6]. The study was approved by the Institutional Review Board.

Radiographic measurement

Preoperatively, anteroposterior (AP) pelvic radiographs were evaluated for cup inclination, anteversion, and leg length discrepancy. Inclination was measured as the angle between a horizontal reference line at the base of the radiographic teardrops and a diagonal line across the rim of the cup. Anteversion was measured by calculating the angle of the ellipse using the Radlink software (El Segundo, CA, USA) on the AP pelvic film. Leg length discrepancy was determined by the vertical distance from a similar point on the lesser trochanters to a horizontal line drawn across the bottom of each radiographic teardrop. Either a cross-table lateral radiograph or a CT scan was qualitatively evaluated to confirm the presence of anterior overhang of the acetabular implant. Postoperatively, radiographs were evaluated for cup inclination, anteversion, and leg length discrepancy. The so-called safe zone for cups was between 30 and 50 degrees of inclination and 10 to 30 degrees of anteversion, according to the Lewinnek definition [7]. The implants were evaluated for loosening, migration, and radiolucent lines, as described previously.

Surgical technique

A DAA was performed on the Hana table, as described by Matta et al. [8]. Dissection was taken to the level of the hip in the standard fashion. Once the modular head was removed, the stem was inspected for stability, position, version, and trunnion condition. As previously mentioned, only hips with well-fixed and well-positioned stems were included in this analysis.

The acetabulum was then exposed by rotating the leg externally and displacing the femoral neck posteriorly. The presence of IPI was confirmed grossly by visualizing the iliopsoas tendon impinging on the anterior metal of the acetabular rim. For cup removal, an acetabular explantation system (Explant Acetabular Cup Removal System; Zimmer Warsaw, IN, USA) was used according to the known size of the cup or from a measurement of the cup’s diameter based on a digital radiograph calibrated with the known femoral head size.

After the remaining bone of the acetabulum was inspected for defects, the acetabulum was spherically reamed medially under fluoroscopic guidance to the radiographic teardrop. Care was taken not to oversize or lateralize the cup. A modular titanium shell was then placed using fluoroscopic imaging to guide abduction and anteversion. After seating, the rim of the cup was palpated to ensure it had been tucked beneath the anterior pelvic rim of the psoas gutter and visualized to confirm the absence of exposed anterior metal rim. Osteophytes were debrided.

The trials were clinically tested for stability, range of motion, and impingement. Intraoperative fluoroscopy was used to assess leg length, offset, and implant position. Adjustments to improve stability were made as needed before the real implants were inserted. As a final step, the insertion of the iliopsoas tendon was partially released at the lesser trochanter to relieve any soft tissue tension with the hip extended and externally rotated.

## Results

Of the eight patients included, there were two women and six men. There were three right and five left rTHAs. The average age of patients at the time of index surgery was 62 years (range: 44-74 years). The mean time to follow-up was 3.3 years (range: 1-9 years).

All eight patients had a prolonged history of groin pain with a mean duration of two years (range: 1 to 4 years) since their primary THA. Seven patients had a positive diagnostic injection with only limited relief therapeutically. One patient declined an injection. No patients had prior surgery for symptoms of IPI.

**Figure 2:**
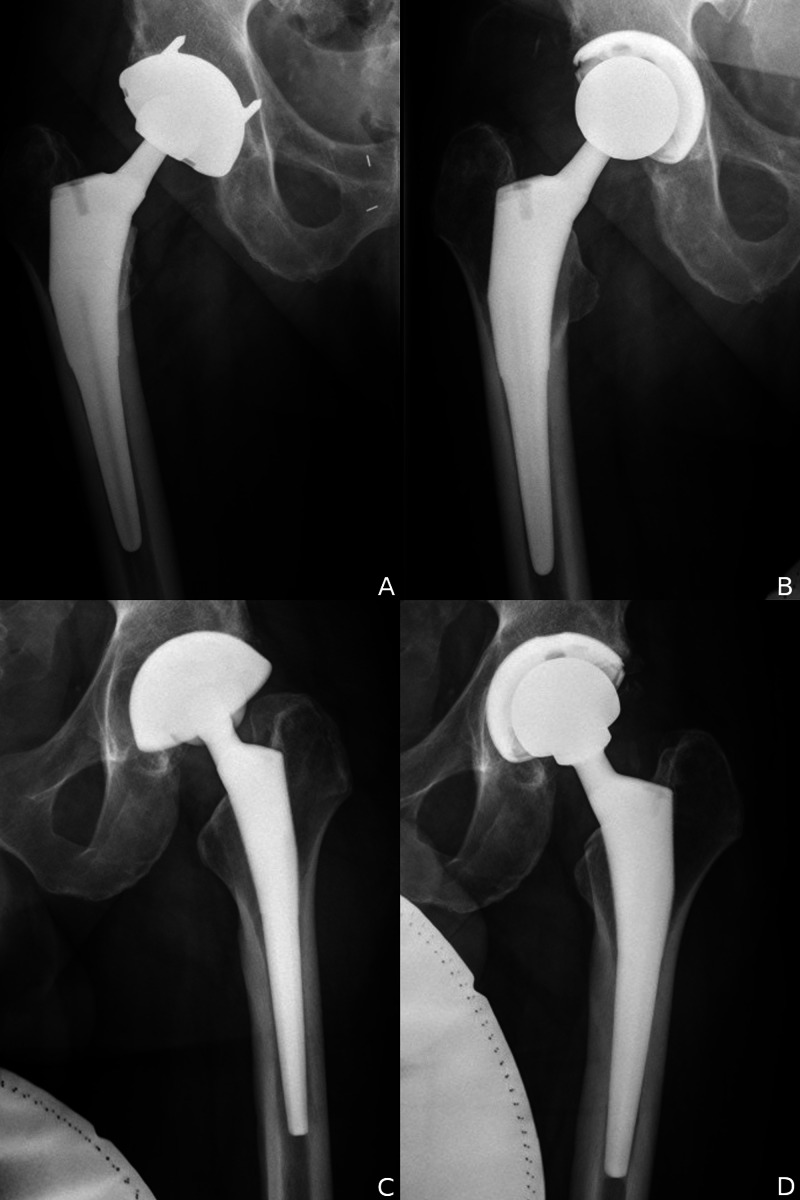
Correction of with cup position and size. (A) Preoperative IPI right hip from lateralized and retroverted cup. (B) Postoperative correction with medialization, anteversion, and downsizing cup. (C) Preoperative IPI of the left hip from lateralized and oversized cup. (D) Postoperative correction with medalization, increased inclination, smaller cup, and larger head size. IPI, iliopsoas impingement

At the time of surgery, the average estimated blood loss was 233 mL (range: 100-300 mL). The explant system was used in all cases without significant bone loss. The remaining acetabular defects in all cases were minimal with an intact rim and could be classified as Paprosky Type I [5]. Preoperatively, the mean cup size was 56 mm (range: 52-60 mm). Postoperatively, the mean cup size was still 56 mm (range: 54-58 mm). Of these, three cups increased by one size, three cups remained the same size, and two cups decreased by one size [Figure 2]. Preoperatively the mean head size was 32 mm (range: 28-36 mm). Postoperatively, six hips had a 40 mm fixed bearing head, one hip had a 36 mm head, and one hip had a 42 mm outer polyethylene ball as part of a dual mobility construct. Cementless acetabular cups were used in all cases. No cages, augments, or allografts were used. Seven cups were stable without screws, and one cup required one screw for adjunctive fixation. No stems were revised nor found to be malpositioned. There were no intraoperative complications. The average length of stay was 1.7 days (range: 1-3 days). All eight patients were discharged home.

Preoperatively, the mean cup anteversion was 4 degrees (range: 0-9 degrees), and the mean cup abduction angle was 41 degrees (range: 22-54 degrees). Postoperatively, the mean cup anteversion was 19 degrees (range: 16-21 degrees), and the mean cup abduction angle was 43 degrees (range: 38-49 degrees). No cups were outside the safe zone. For limb length inequality, the average amount of preoperative discrepancy was 7 mm (range: 2-13 mm). The average amount of postoperative limb length discrepancy was 4 mm (range: 0-10 mm). At the final follow-up, all hips remained radiographically stable.

All patients regained strength in hip flexion with a normal straight leg raise by six months. At follow-up, the mean HOOS, Jr score was 75 points (range: 32-100 points). The patient with a score of 32 also had severe ongoing back pain and was awaiting a spinal cord stimulator. Given the recent transition to patient-reported outcomes, a preoperative HOOS, Jr score was not available for comparison. There were no reported revisions or reoperations.

## Discussion

This intent of acetabular revision with a partial iliopsoas tenotomy through a DAA is to mitigate surgical risks. Although rTHA for IPI is associated with a significant rate of complications, the uncertainties in this series were well-managed. There were no postoperative dislocations, fractures, or revisions. The combination of clear indications, intraoperative imaging, an acetabular explant system, and a partial iliopsoas tenotomy under direct visualization from a DAA may have contributed to these more favorable outcomes.

**Figure 3:**
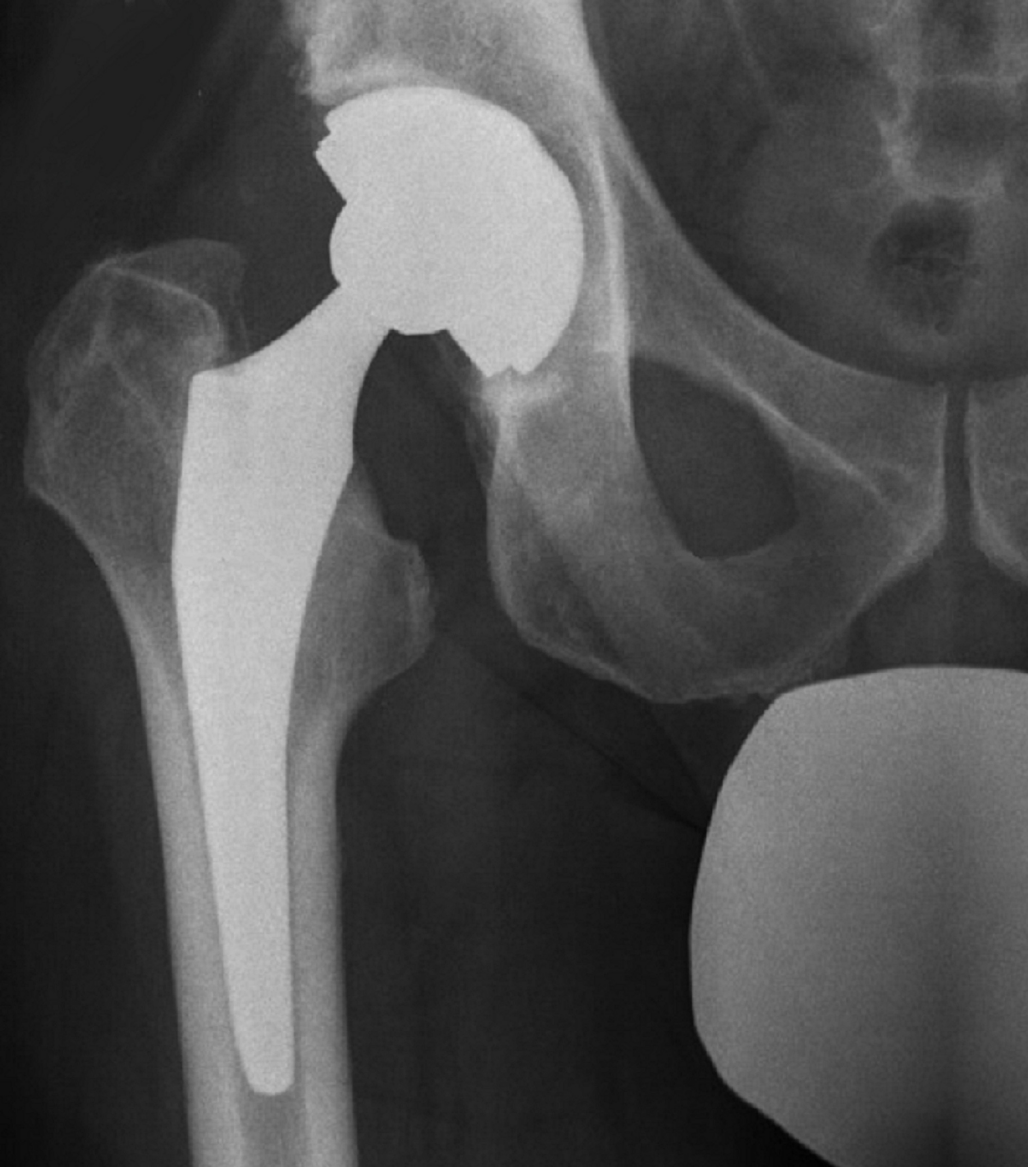
Typical radiographic appearance of IPI on AP pelvic film with insufficient anteversion of the acetabular component. Note the absence of radiographic ellipse at the cup opening. IPI, iliopsoas impingement; AP, anteroposterior

The patients in our series complained of a frustratingly prolonged history of groin pain before receiving a diagnosis. Groin pain had always been refractory to conservative measures, and at times unresponsive to multiple rounds of physical therapy and anti-inflammatories. The most striking findings were a weakness in the straight leg raise, known as a positive Stinchfield test, and temporary relief after an image-guided injection. An AP radiograph of the hip would then uniformly reveal an acetabular implant lacking sufficient anteversion [Figure 3]. In the first several cases, anterior overhang of the cup was further confirmed on the cross-table lateral view. In later cases, we turned to CT scans with three-dimensional reconstructions to define the area of anterior impingement. Due to the artifact, we were unable to interpret the magnetic resonance imaging findings brought to our office. In retrospect, the advanced imaging modalities did not change our management. In recognizing the difficulty in diagnosis, we now rely on a positive Stinchfield test, a positive diagnostic iliopsoas injection, and a positive cross-table lateral view.

**Figure 4:**
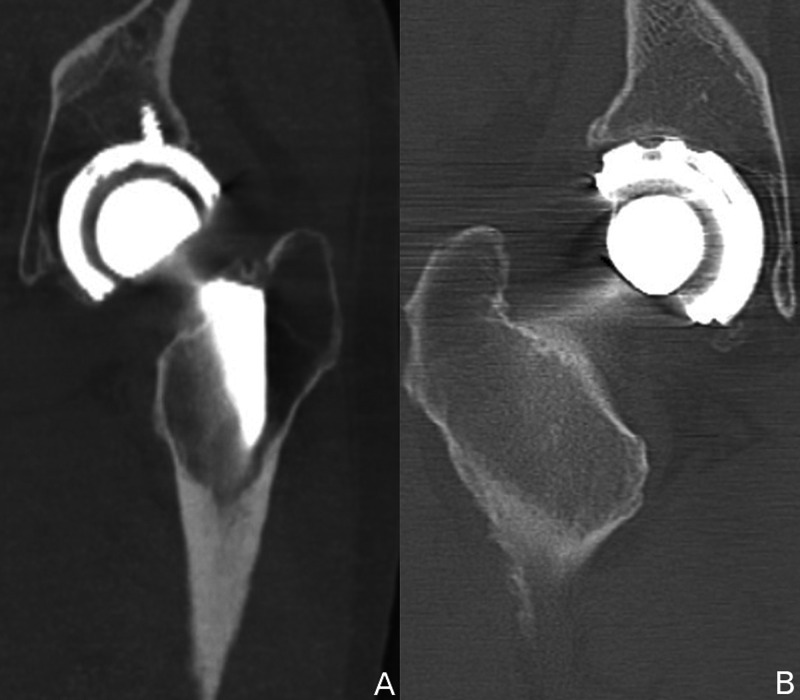
Excessive cup lateralization contributing to IPI. (A) Coronal CT scan of the left hip showing cup placement lateral to the base of the fossa. (B) Coronal CT scan of the right hip showing vertical cup and lateral placement relative to the fossa. IPI, iliopsoas impingement; CT, computed tomography

**Figure 5:**
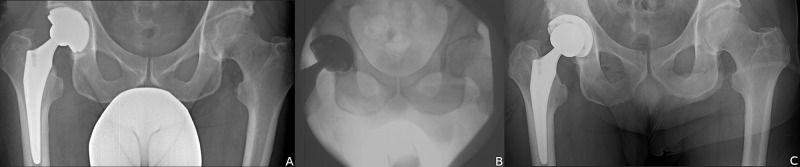
Management of IPI with cup repositioning. (A) Preoperative AP radiograph with vertical and retroverted cup. (B) Intraoperative imaging to confirm cup inclination and version. (C) Follow-up radiograph with well-fixed components and improved cup orientation. IPI, iliopsoas impingement; AP, anteroposterior

The etiology in all our cases was related to acetabular implant malposition. Like others, we found the acetabular implant to be insufficiently anteverted, excessively lateralized, or both [Figure 4]. In these positions, the anterior edge of the cup extends beyond the pelvic rim, specifically located where the iliopsoas exits the psoas gutter. Dora et al. noted that all 22 hips undergoing revision had the same pathologic prominence of the anterior cup [9]. Schoof et al. found that 12 out of 12 cups were either neutral or retroverted, and that of these 12 of 11 cups were also overstuffed [3]. In a review of IPI, Lachiewicz and Kauk proposed, “acetabular revision is usually recommended when the preoperative plain radiographs or CT scan demonstrate that the anterior edge of the acetabular component protrudes in front of the anterior bony acetabular rim” [10]. In recognizing the causal etiology of implant position, we also elected to proceed with cup repositioning rather than implant retention with tenotomy alone [Figure 5].

The controversy of tenotomy with implant retention versus acetabular revision lies in the possibility of more frequent and severe complications with the latter. After a consecutive series of 30 hips with IPI, Dora et al. warned that tenotomy was preferable mostly to avoid the risks of revision [9]. The fundamental technical challenge lies in the removal of a well-fixed cup with as minimal bone loss as possible. Otherwise, the magnitude of the revision can escalate rapidly. In their first case, Schoof et al. encountered “extended” bone loss that required stabilization with a Burch-Schneider cage and allograft [3]. We also agree that the most precarious portion of the procedure is cup removal even with an explant system. Helpfully, the thin blades are far more conservative than conventional curved chisels, and correct sizing of the blades is established by calibrating the cup diameter from a known head size on the radiographs. The anatomic window of the DAA did not compromise visualization relative to a posterior approach in our experience. Rather, it provided direct access to the area of pathology and facilitated insertion of the explant blades along the anterior rim.

**Figure 6:**
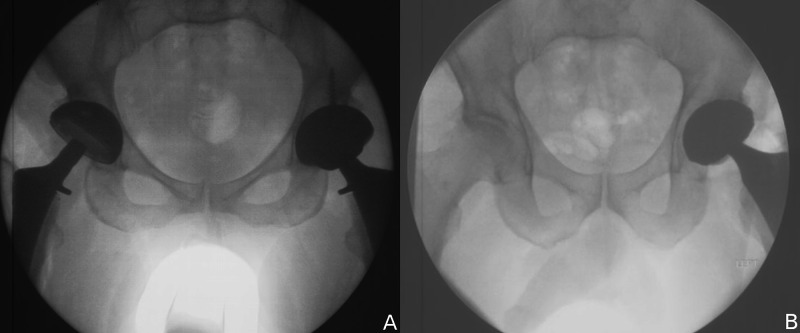
Intraoperative imaging to guide implant position. (A) Right hip imaging with corrected acteabular position. (B) Left hip imaging confirming leg length and offset symmetry with the final implant in place.

Cup size and position were guided by the use of intraoperative imaging. The first stage of imaging occurred during reaming. In cups that had been excessively lateralized, fluoroscopic imaging was used to guide the reamer to the teardrop radiographically and to the floor of the cotyloid notch anatomically. Our next objective was to avoid oversizing the cup. It is not uncommon to rely on a jumbo cup for revisions using a cup at least 10 mm larger than the average [11]. In this series, we deliberately limited an increase in cup size to avoid “overstuffing,” as described by Schoof et al. [3]. Consequently, the mean preoperative cup diameter did not increase. While three cups increased by 2 mm in cup diameter, the other five cups were the same size or smaller than the original implant. Finally, conventional alignment guides and intraoperative imaging were used to correct cup abduction and anteversion. Small adjustments to version were often necessary to eliminate any metal prominence at the anterior pelvic rim before the final seating [Figure 6]. All revised cups were placed in the so-called safe zone [7].

Although iliopsoas tenotomy has been described as an alternative to revision, we have viewed it as an adjunct to revision. Given the small series and the combination of the two techniques, it is difficult to attribute the improvement in clinical outcomes to either tenotomy or cup repositioning. With the DAA, however, a choice between the two techniques does not have to be made. The iliopsoas insertion at the base of the lesser trochanter is easily exposed from the front with external rotation of the femur. Further, the extent of partial release can be adjusted by palpating the tension with the leg extended and externally rotated.

The limitation of this study is that it is a single surgeon, small retrospective series. Larger numbers of patients and longer follow-up may reveal a complication profile similar to that of other series. Also, we were only able to identify for analysis those patients who had undergone revision surgery for IPI, and hence, we cannot comment on those who improved with conservative treatment or those who chose to proceed with arthroscopic tenotomy elsewhere. Alternatively, an algorithm for either tenotomy or revision based on the amount of overhang could also be followed, as proposed by Chalmers et al. [12]. In hindsight, a postoperative cross-table lateral film would have been helpful to confirm appropriate cup position but was not included as a routine part of the follow-up radiographic examination. Finally, with the recent transition to patient-reported outcomes using the HOOS, Jr score, a preoperative score was not available for comparison.

## Conclusions

We agree with other studies that rTHA is a solution of last resort. Conservative measures should be exhausted before considering rTHA. Our patients typically presented for multiple preoperative visits to review prior failed treatment. In the event that surgery is indicated, we recognize that arthroscopic tenotomy has been met with clinical success and could be a first surgical step. Though we have little familiarity with the procedure, we foresee that it would be unlikely to compromise an open revision should the former fail clinically. Our experience is far too limited to know which treatment protocol is best. We do know, however, that we did not encounter the complications reported in larger series and that we continue to appreciate the risks of the procedure.
